# 46 Burn-related Violence Against Women in the US: Findings from the ABA Burn Registry

**DOI:** 10.1093/jbcr/irae036.070

**Published:** 2024-04-17

**Authors:** Colton Wayne, Claudia C Malic, Yvonne M Singer, Nicole P Bernal

**Affiliations:** Nationwide Children’s Hospital, Columbus, Ohio; CHEO, Ottawa, ON; Griffith University, Melbourne, Victoria; The Ohio State University Wexner Medical Center, Columbus, OH; Nationwide Children’s Hospital, Columbus, Ohio; CHEO, Ottawa, ON; Griffith University, Melbourne, Victoria; The Ohio State University Wexner Medical Center, Columbus, OH; Nationwide Children’s Hospital, Columbus, Ohio; CHEO, Ottawa, ON; Griffith University, Melbourne, Victoria; The Ohio State University Wexner Medical Center, Columbus, OH; Nationwide Children’s Hospital, Columbus, Ohio; CHEO, Ottawa, ON; Griffith University, Melbourne, Victoria; The Ohio State University Wexner Medical Center, Columbus, OH

## Abstract

**Introduction:**

Violence against women is a global public health problem and a violation of human rights. CDC data shows 41% of US women have experienced intimate partner violence. Burn-related violence against women (BVAW) is an extremely confronting form of physical violence, however, limited evidence has been published in scientific literature. The aim of this study was to describe the frequency, demographic, injury and outcome characteristics of women admitted to US burn centers who have experienced burn violence and determine if these characteristics were different to characteristics of women admitted with accidental burns.

**Methods:**

Data was extracted from the ABA National Burn Registry (NBR) for women, ≥ 18 years old, admitted to US Burn Centers between 2008 and 2018. NBR injury intent variables identified women who sustained burn violence or accidental injuries to separate them into two comparator groups. All NBR ‘accidental’ injury intent variables were collapsed into one variable. Women who experienced self-harm were excluded. Descriptive and simple comparative statistics were used to describe and compare groups.

**Results:**

There were 54,630 women who met study inclusion criteria, 1063 (2%) experienced burn violence. Women who experienced burn violence were younger (38.1 yrs [mean]) than women with accidental injuries (47.8 yrs). A significantly greater proportion of the burn violence group were Black/African American women compared to the accidental injury group (44.7% vs. 22.1%, p< 0.05) and a greater proportion of burn violence patients were covered by Medicaid (40.1% vs. 22.1%, p< 0.05). Women who sustained violence had more severe injuries than their counterparts, in terms of both %TBSA extent ([median] 6.1% vs. 3.0%, p< 0.05) and burn depth (3rd degree) ([median] 11.0% vs. 2.0 %, p< 0.05). Scald and flame injuries were the most frequent mechanism of injury in both groups. Length of hospital stay was longer for women who sustained burn violence ([median] 15.9 vs. 9.2 days, p< 0.05) and ICU stay ([median] 21.0 vs. 6.5 days, p< 0.05) was also significantly longer. However, there were no differences in mortality rates between groups (5.5% vs 4.3%).

**Conclusions:**

Whilst the frequency of women who sustained burn violence, and were admitted to US burn centers appears small, they are confronting injuries that can result in significant physical and psychological sequelae. Compared to women with accidental burns, women who experience burn violence were younger, disproportionately Black/African American, suffered more severe injuries and experienced poorer outcomes. American women deserve better

**Applicability of Research to Practice:**

Burns can be easily explained as unintentional, especially when women can’t tell their stories. To protect women, US burn centers require robust, standardized approaches to identify and respond to burn violence and the ABA can lend a voice to advocacy efforts against violence against women, collaborating alongside stakeholder organizations.

